# IMAT-IGRT Treatment with Simultaneous Integrated Boost as Dose Escalation for Patients with Cervical Cancer: A Single Institution, Prospective Pilot Study

**DOI:** 10.3389/pore.2021.608446

**Published:** 2021-03-24

**Authors:** Zoltán Lőcsei, Klára Sebestyén, Zsolt Sebestyén, Eszter Fehér, Dorottya Soltész, Zoltán Musch, László Csaba Mangel

**Affiliations:** ^1^ Department of Oncotherapy, Clinical Center, University of Pécs, Pécs, Hungary; ^2^ Pécs Diagnostic Center, Pécs, Hungary

**Keywords:** cervical cancer, SIB, IGRT, IMRT, dose escalation, toxicity 3

## Abstract

**Purpose:** The aim of this study was to introduce the simultaneous integrated boost (SIB) technique to assess the safety of replacement of the brachytherapy (BT) boost for ineligible patients with cervical cancer receiving radiochemotherapy (RCT).

**Methods:** Fourteen patients were enrolled between 2015 and 2018. SIB was delivered using RapidArc technique at doses of 2.4 Gy per fraction during pelvic irradiation with 50.4/1.8 Gy in seven patients (to a total dose of 67.2 Gy) with limited volume disease. In 7 patients with a more advanced disease stage (>5 cm tumor, parametric invasion both sides), parametric boost therapy was added to the pelvic radiotherapy to a total dose of the macroscopic tumor of 79.2 Gy. All patients received simultaneous cisplatin-based chemotherapy for 5 cycles with a dosage of 40 mg/m^2^. We examined acute toxicity (CTCAE v4.1) and quality of life (EORTC QLQ30 and CX24). The tumor regression rate was evaluated with RECIST 1.1 after the first 3- to 4-months follow-up Magnetic Resonance Imaging (MRI) scan. We calculated the percentage of tumor regression rate and the local control during the follow-up period and evaluated the survival data.

**Results:** Our patient data are presented at a median follow-up time of 24.5 months. During the treatment period, no grade 3 to 4 toxicity was observed. During the follow-up period, no late-onset toxicity was observed. The tumor regression rate at the first MRI scan was 95.31% on average. Disease free survival (DFS) during the median follow-up of 24 months was 98.6%.

**Conclusion:** In patients with cervical cancer, the SIB technique is amenable as part of definitive RCT. Dose escalation with the SIB technique can be safely administered to cervical cancer patients during definitive RCT if BT is not feasible. However, further randomized clinical studies are needed to validate the method, so routine use of it cannot be recommended yet.

## Background

Cervical cancer accounts for 30% of all gynecological malignancies in the developed world. The pathogenic role of HPV infection in the development of cervical cancer is well known with HPV subtypes 16, 18, 31, 33 and 45 considering highly pathogenic. The macroscopic spread of the tumor is characterized according to FIGO and TNM classifications. Patients with cervical cancer classified as more advanced than FIGO stage Ib2 and stage T1b2 generally receive definitive radiochemotherapy (RCT). In these stages, treatment is performed most commonly using RCT as follows: percutaneous pelvic radiotherapy (RT) at a dose of 45 to 50.4 Gy using the 4-field box or IMRT (intensity modulated RT) technique at daily doses of 1.8 Gy per fraction, and pathological lymph nodes are boosted with SIB to 61,2 Gy with 2,2 Gy fractions, simultaneously with cisplatin chemotherapy at doses of 40 mg/m^2^ weekly in 4 to 5 cycles. According to the globally approved “gold standard,” 3 to 4 x 7 Gy image based intracavitary HDR-AL boost irradiation is performed at the end of the treatment. If the lesion is large combined intracavitary/interstitial implant is recomended [1, 2].

However, in a proportion of the patients, brachytherapy (BT) is not feasible (due to anatomical factors, extreme scarring etc.), or some patients do not consent to the procedure. BT may also be limited by the inability of intracavitary techniques to provide appropriate tumor coverage. However, thanks to the impressive progress in teletherapy technology by the routine use of IGRT (Image Guided RT), IMRT and IMAT (Intensity Modulated Arc Therapy) methods, the possibility of developing an alternative external beam dose escalation model may also arise. With external beam radiotherapy using the SIB (simultaneous integrated boost) technique, this type of RCT is theoretically feasible. The aim of our study is to develop an alternative treatment method for our patients which is safe and is not different from the standard therapy in terms of its effect. In our present paper, we would like to share our first results.

## Patients and Methods

Our study was performed according to the approval of the ETT TUKEB (Medical Research Council - Scientific and Research Ethics Committee) registration number 5620–3/2015/EKU on 21/01/2015 and the Regional Research Ethics Committee of the University of Pécs registration number. 5,409. The study was registered to the German Clinical Trial Platform on 13/11/19 with the registration number DRKS00019044. In our study, patients with FIGO stages from IIB to IVA were enrolled. Patient enrollment was performed between January 2015 and September 2018, in which period 14 patients were treated. Most of the patients do not consent to BT due to psychological factors, BT was not feasible due to bladder involvement, or extreme obesity. Their median age was 57.5 years (37 to 78). All procedures were performed in accordance with the relevant guidelines and regulations. All patients signed an informed consent form before study enrollment. The TNM classifications of the treated cervical cancers were stage from T2b to T4. In addition, lymph node involvement was observed in 9 patients (Table 1.).

**TABLE 1:** Patient characteristics.

	SIB-Dose	TNM	Stage	Age
PT-001	67.2 Gy	T4N0M	0 IVA	78
PT-002	67.2 Gy	T2bN0M0	IIB	60
PT-003	67.2 Gy	T2bN0M0	IIB	60
PT-004	67.2 Gy	T2bN1M0	IVA	55
PT-005	79.2 Gy	T4N0M0	IVA	37
PT-006	79.2 Gy	T4N1M0	IVA	44
PT-007	67.2 Gy	T2bN1M0	IVA	40
PT-008	79.2 Gy	T4N1M0	IVA	49
PT-009	79.2 Gy	T3bN1M0	IVA	46
PT-010	79.2 Gy	T4N1M0	IVA	43
PT-011	79.2 Gy	T3bN0M0	IIIB	66
PT-012	79.2 Gy	T3bN1M0	IVA	70
PT-013	67.2 Gy	T2bN1M0	IVA	75
PT-014	67.2 Gy	T4N1M0	IVA	75

### Diagnostics

The generally applied physical examination was supplemented by an MRI scan within 6 weeks prior to treatment for the purpose of a more exact staging. Prior to the initiation of percutaneous RT, PET/CT scans were performed in radiotherapy treatment position to help to determine the exact GTV (“gross tumor volume”).

### Target Volume Delineation

For the determination of the GTV-T, we used 3 noncontrast planning CT scans with 3-mm thickness. The first scan was performed with a full bladder, and the second scan with an empty bladder. After the second scan, the patient drank 300 ml water, and the third scan was performed after half an hour. An iodine-marked tampon was inserted in the patient’s vagina for all 3 scans to mark the bottom of the cervix. The T1, T2 and MPRAGE contrast-enhanced MRI sequences and the PET/CT scans were deformable registered to the planning CT. In all images, we defined the GTV-T and created an SIB-GTV as an ITV (internal target volume). Using a 3-mm safety margin, we created the SIB-PTV (planning target volume).

A CTV-N (clinical target volume of nodes) was determined according to institutional and RTOG protocols that included the mentioned SIB-GTV. We used a 5-mm margin for the pelvic PTV.

### Treatment Planning

Planning was performed on the third planning CT. The treatments were performed with RapidArc technique using a Novalis TX linear accelerator.

In seven patients in whom the primary tumor was less than 5 cm in longest diameter as measured by MRI, SIB was delivered in 28 fractions at doses of 2.4 Gy per fraction during pelvic irradiation with a total dose of 50.4/1.8 Gy (the total dose to PTV-SIB was 67.2 Gy). Thus, the BED (biologically effective dose) of the dose delivered to the cervical tumor was 83.33 Gy, which was calculated with an α/β value of 10. During the treatment, the dose limits of the organs at risk (OAR) applied in BT has been used: rectal wall D2cc, 64 Gy; bladder wall D2cc, 85 Gy; sigmoid colon wall D2cc, 63 Gy, respectively. The treatment with SIB represents with an α/β value of 3 a BED of 120,96 and 142.56 Gy. During the determination of normal tissue tolerance doses, no further biological conversion was performed, because to the similarity of doses reached in BT.

In another seven patients in whom the size of the cervical cancer exceeded 5 cm in longest diameter and the parametrium was invaded on both sides, a higher final dose and expanded target volume were used based on the local protocol. A further 10-Gy dose of parametrial boost was added to the pelvic RT after performing a new simulation CT for replanning procedure, while the treatment of the primary tumor was continued with doses of 2.4 Gy per fraction (the total dose to PTV-SIB was 79.2 Gy). The dose of 2.4 Gy delivered to 33 fractions corresponds to a BED of 98.21 Gy, with an α/β value of 10. The dose limits for the normal tissue were taken into account as detailed above.

### Treatment Delivery

During the treatments and prior to the planning CTs, the patients were provided with strict dietary recommendations. Under the increased control of the volume and position of the bladder and the rectum, SIB-PTV was minimized by decreasing the displacement of the cervix. Using the ITV concept, we were able to compensate for the motion of the target area, thereby keeping the final PTV as small as possible. The irradiation treatment was on line controlled using 3D cone beam CT five times during the first week of the treatment and then once a week according to an offline treatment protocol. The dietary protocol was in place that eliminates foods that cause bloating. Simultaneous weekly cisplatin was administered to all patients at a dosage of 40 mg/m^2^. All patients were treated with 5 cycles of chemotherapy. In only 4 cases, we observed Gr I-II neutropenia when granulocyte stimulating factor needed to be administered for secondary prevention, according to institutional protocol.

### Quality of Life and Follow up

The primary endpoint of the study was acute toxicity and quality of life (QoL), for which the EORTC-QLQ 30 and CX-24 questionnaires were used. The patient completed the questionnaire on the first treatment day and on the third and fifth weeks of treatment. Weekly physician visits were performed to adjust adverse events with CTCAE grading and indicate supportive treatment if needed. After treatment completion monthly physician visits were performed in the first 6 months, then every 3 months. Primarily the main side effects, like diarrhea, vomiting, vaginal bleeding or discomfort were collected. No QoL questionnaires were performed during the follow-up period. The tumor regression rate was determined based on the first 3–4 months of follow-up MRI scan by measuring the primary tumor and lymph node metastasis reduction in the size of the longest diameter in accordance with the RECIST 1.1 (Response Evaluation in Solid Tumors) criteria. RECIST 1.1 radiological evaluation was performed on the same MRI machine with the same pelvis protocol. The percentage of tumor shrinkage for the whole population was calculated. The secondary endpoint was to collect our patients’ survival data during the follow-up period exceeding the median value of 24 months. If 2 consecutive MRI scans confirmed residual mass, a PET/CT scan was performed to confirm residual tumor activity.

## Results

The tumor stage was IVA in 78%, IIIB in 7%, and IIB in 14%, respectively. The average tumor diameter was 48.7 mm (22–83 mm) at the start of treatment, as measured on axial MRI. During our study, grade 3 to 4 toxicity was not observed, which provided a kind of a positive answer in terms of the feasibility and tolerability of the treatment. Longer than 3 days treatment interruption was not needed. The two SIB dosage schemes could be safely implemented. The doses regarding the organs at risk were maintained as originally planned.

### Dose Exposure for Organs at Risk

The dose exposure of the bladder could be maintained at the mean and maximum doses as well as at the D2cc values in accordance with the literature data. The V50 values were on average higher than the standard, but the V70 values were markedly lower than expected (Table 2.).

**TABLE 2:** Bladder doses for all patients.

Bladder	Mean (Gy)	MAX (Gy)	D2cc(Gy)	V50(%)	V70 (%)
All Cases	48.09	72.00	67.30	46.20	10.84
67.2 Gy	44.08	68.34	64.39	27.87	0.00
79.2 Gy	52.11	75.66	70.21	64.53	10.84

In the case of the rectum, the maximum and D2cc values were higher than the accepted dose limits of the HDR-AL technique, but the V50 and V70 percentages were well within the acceptable limits (Table 3).

**TABLE 3:** Rectum doses for all patients.

Rectum	Mean (Gy)	MAX (Gy)	D2cc(Gy)	V50(%)	V70 (%)
All Cases	49.77	72.89	68.25	42.29	10.34
67.2 Gy	46.09	68.99	64.17	32.17	0.00
79.2 Gy	53.44	76.78	72.33	52.41	10.34

In the case of the sigmoid colon, a dose which may be associated with an increased risk of adverse reactions was not observed, and the average D2cc was between 62 and 67 Gy (Table 4.).

**TABLE 4:** Sigma doses for all patients.

Sigma	Mean (Gy)	MAX (Gy)	D2cc(Gy)
All Cases	52.57	70.29	62.90
67.2 Gy	47.30	63.03	54.52
79.2 Gy	55.73	74.65	67.92

### Toxicity and Quality of Life

**FIGURE 1:**
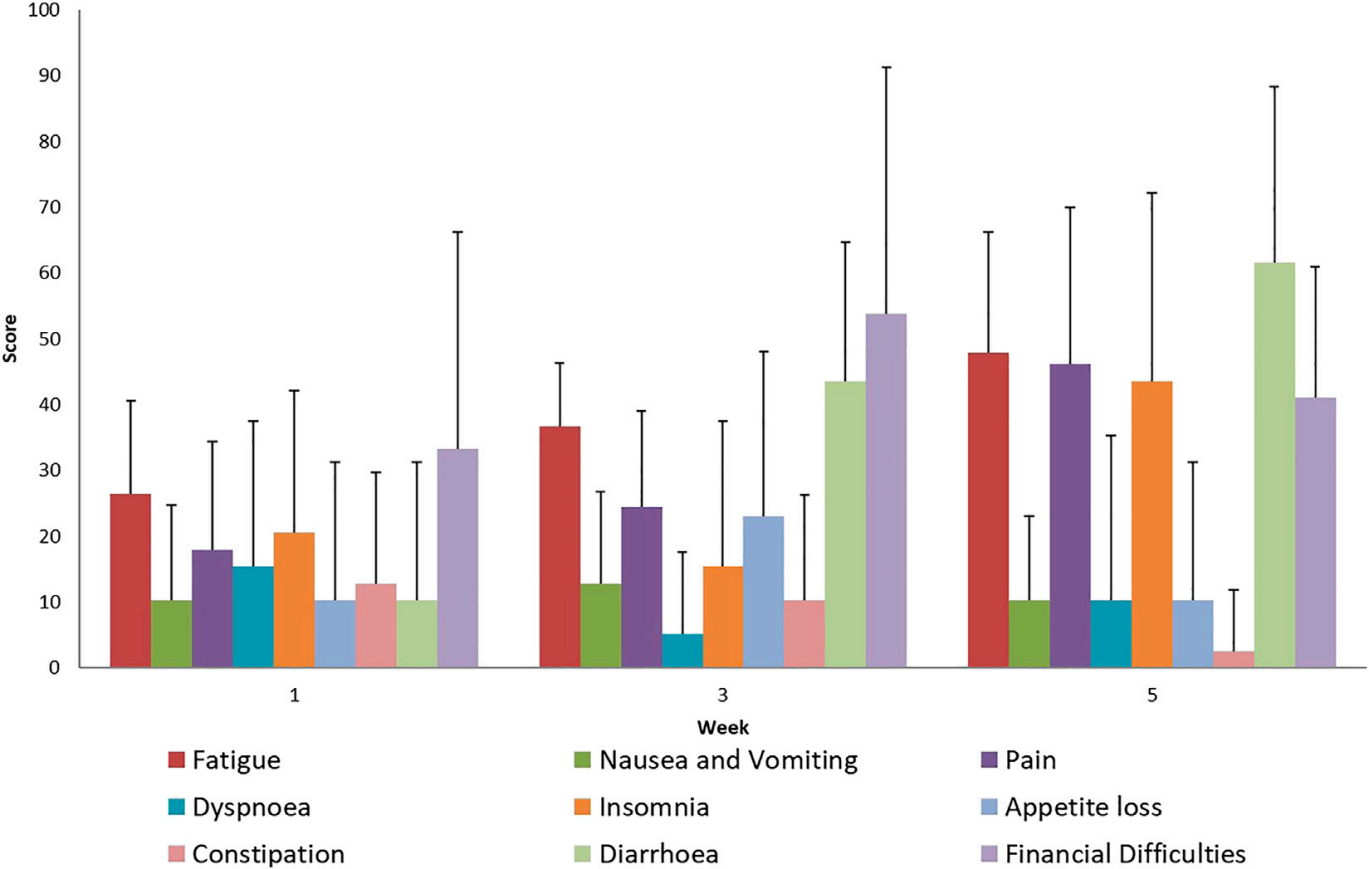
Evaluation of EORTC QLQ-C30 Symptoms questionnaires score mean value and SD (+), during treatment for all patients.

**FIGURE 2:**
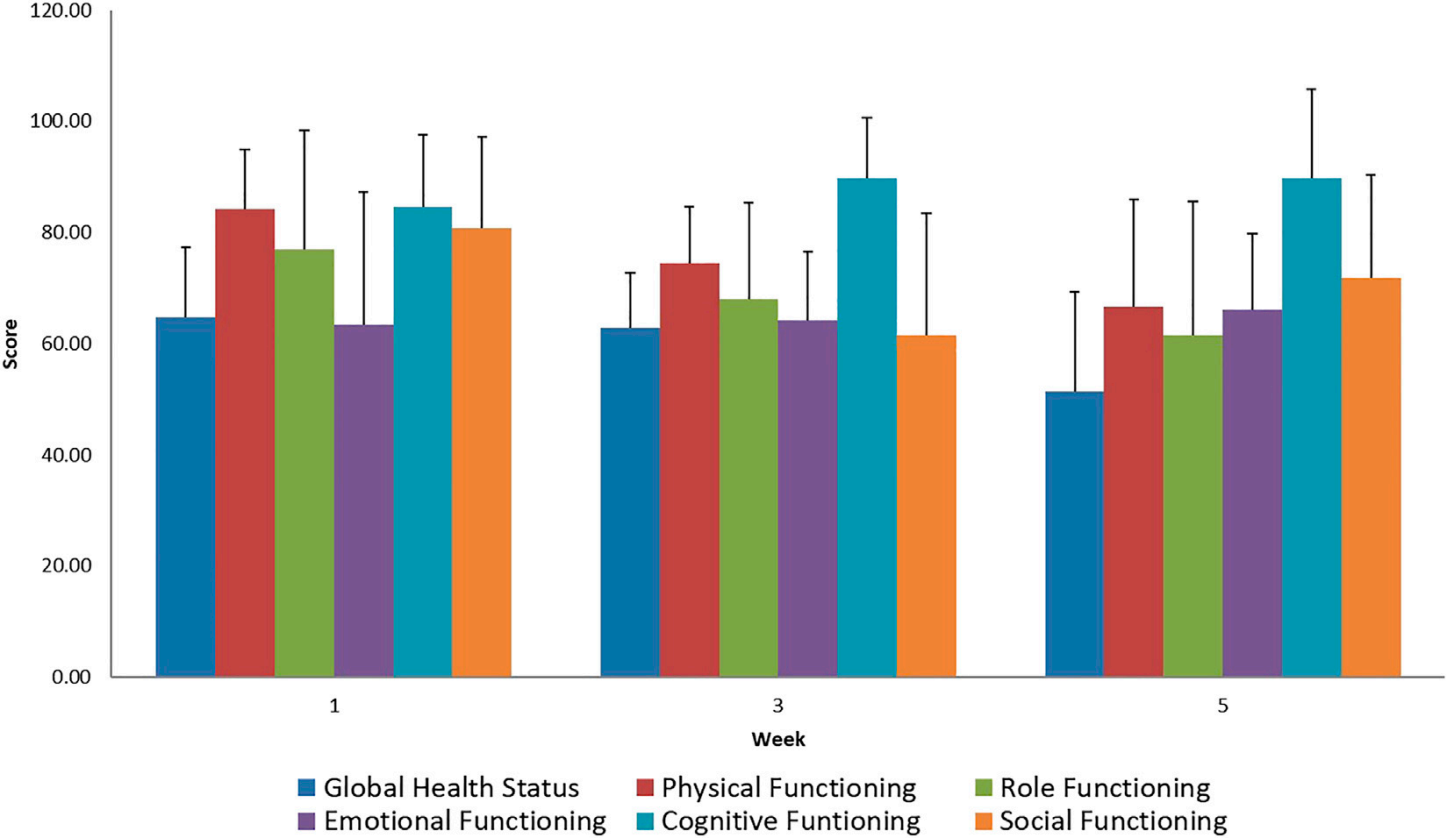
Evaluation of EORTC QLQ-C30 Functional Scale questionnaires score mean value and SD (+), during treatment for all patients.

During the treatment, adverse reactions were assessed by EORTC questionnaires and weekly physician visits. The results of these studies confirmed the usual complaints, such as diarrhea, bladder inflammation, and nausea, associated with intensive combined therapy. During the evaluation of the well-being scales, in the case of the EORTC QLQ-C30 questionnaire, worsening social life could be observed, which can be explained by the symptoms related to the treatment (Figures 1, 2). The complaints of diarrhea, fatigue, insomnia and pain as measured on the symptom scale of the questionnaire became aggravated. However, similar adverse reactions also occur during conventional treatments. No acute adverse reactions above Grade 2 occurred. Currently, at the two-year follow-up, severe proctitis or bladder stricture have not developed. DFS was calculated from the percentage of patients without determined cancer. No further statistical statement could be done due to the small cohort size of our patients.

**FIGURE 3:**
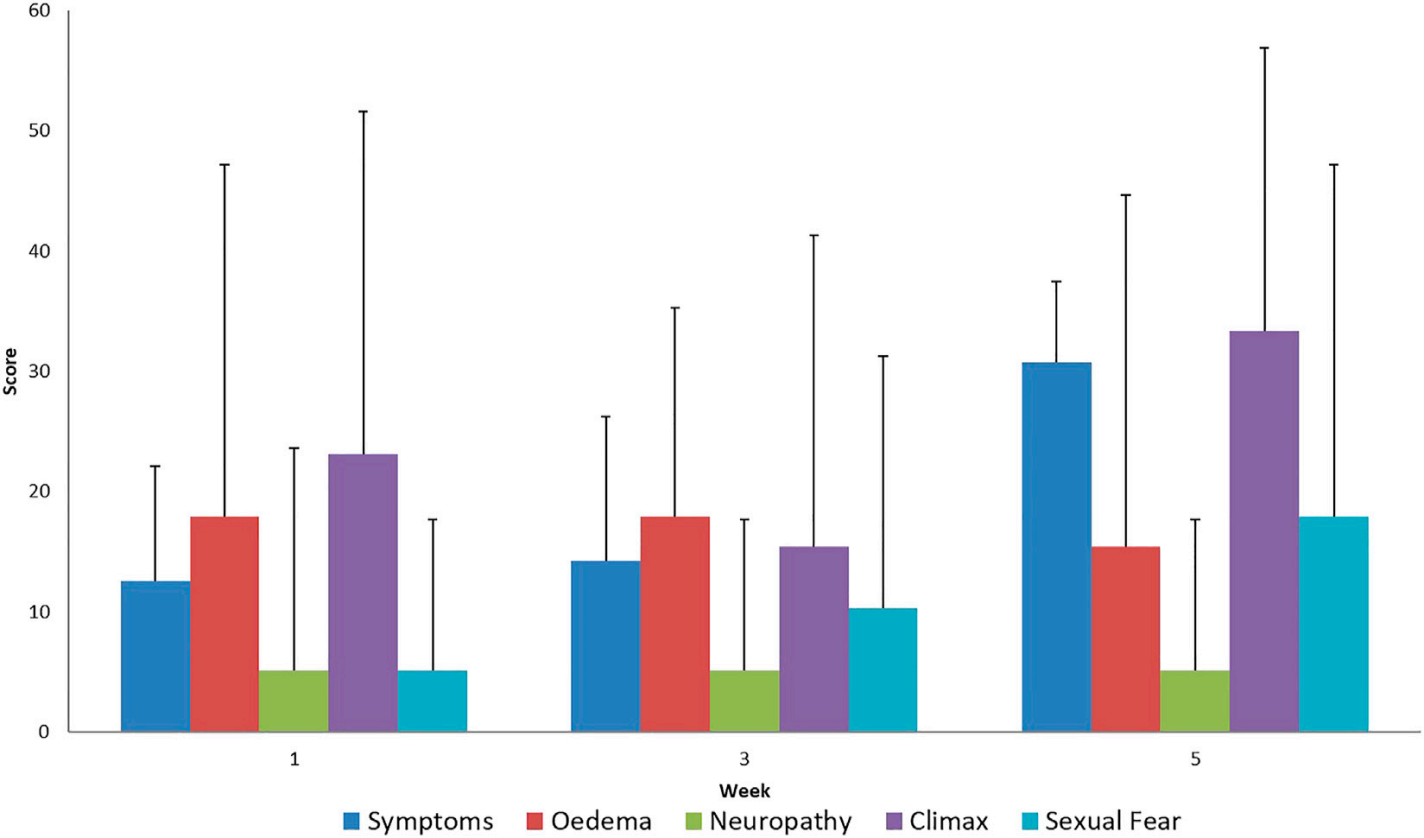
Evaluation of EORTC QLQ-CX24 Symptoms questionnaires score mean value and SD (+), during treatment for all patients.

**FIGURE 4:**
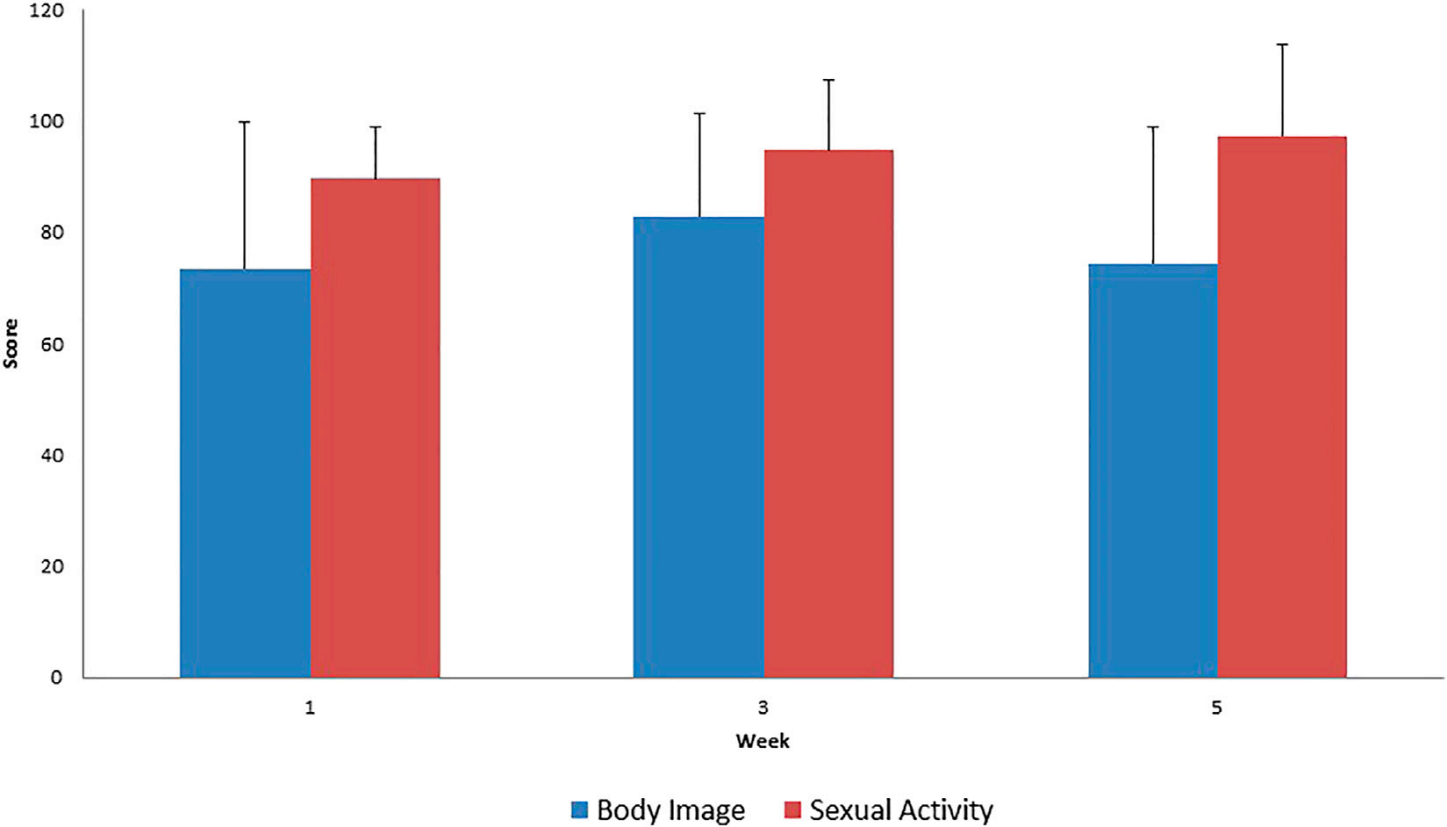
Evaluation of EORTC QLQ-CX24 Functional Scale questionnaires score mean value and SD (+), during treatment for all patients.

In the disease-specific EORTC QLQ CX 24 questionnaires, the aggravation of menopause-like complaints was considered normal since it is a common adverse reaction in the case of pelvic radiotherapy (Figures 3, 4). Unfortunately, a very low percentage of our patients provided meaningful responses to questions about body image and sexuality possibly due to reasons associated with pudency.

During the evaluation of the EORTC QLQ-C30 questionnaire regarding general symptoms, we noted an increase in the general adverse reactions experienced during the treatment. Minimal loss of appetite and the decrease in nausea suggest the use of appropriate supportive therapy. A decreased degree of participation in social activities while battling the disease is acceptable during therapy.

The results of EORTC QLQ-CX24 functional scale questionnaire are difficult to interpret because a low willingness to respond was noted during the evaluation of the questionnaire. Thus, the absence of actual changes in sexual activity and body image is questionable.

Supporting the QLQ-C30 questionnaire results, an increase in treatment related symptoms was also observed in the tumor-specific questionnaire. The increase in menopause-like symptoms as a result of iatrogenic infertility caused by radiotherapy is normal side effect and is not specifically associated with the delivery of the SIB dose.

### Follow Up Data

We present our data at a median follow-up time of 24.5 months (in a range of 9 to 45 months).

Based on the 3-months follow-up MRI scans after the treatment, an average of 95.31% regression rate was measured in terms of tumor size reduction. Complete response was achieved in 10 patients, and partial response was achieved in 4 patients (Table 5).

**TABLE 5:** Evaluation of the treatment.

	PreTreatment MR Size (mm)	PostTreatment MR Size (mm)	RECIST	Regression
PT-001	25	0	CR	100%
PT-002	22	0	CR	100%
PT-003	47	0	CR	100%
PT-004	42	0	CR	100%
PT-005	80	3	PR	96%
PT-006	59	4	PR	91%
PT-007	49	30	PR	67%
PT-008	85	0	CR	100%
PT-009	52	0	CR	100%
PT-0010	60	0	CR	100%
PT-0011	82	18	PR	65%
PT-0012	67	0	CR	100%
PT-0013	35	0	CR	100%
PT-0014	38	0	CR	100%

CR: Complete Response, PR: Partial Response.

After the median 24-months follow-up, the actuarial DFS value was 98.6%.

**FIGURE 5:**
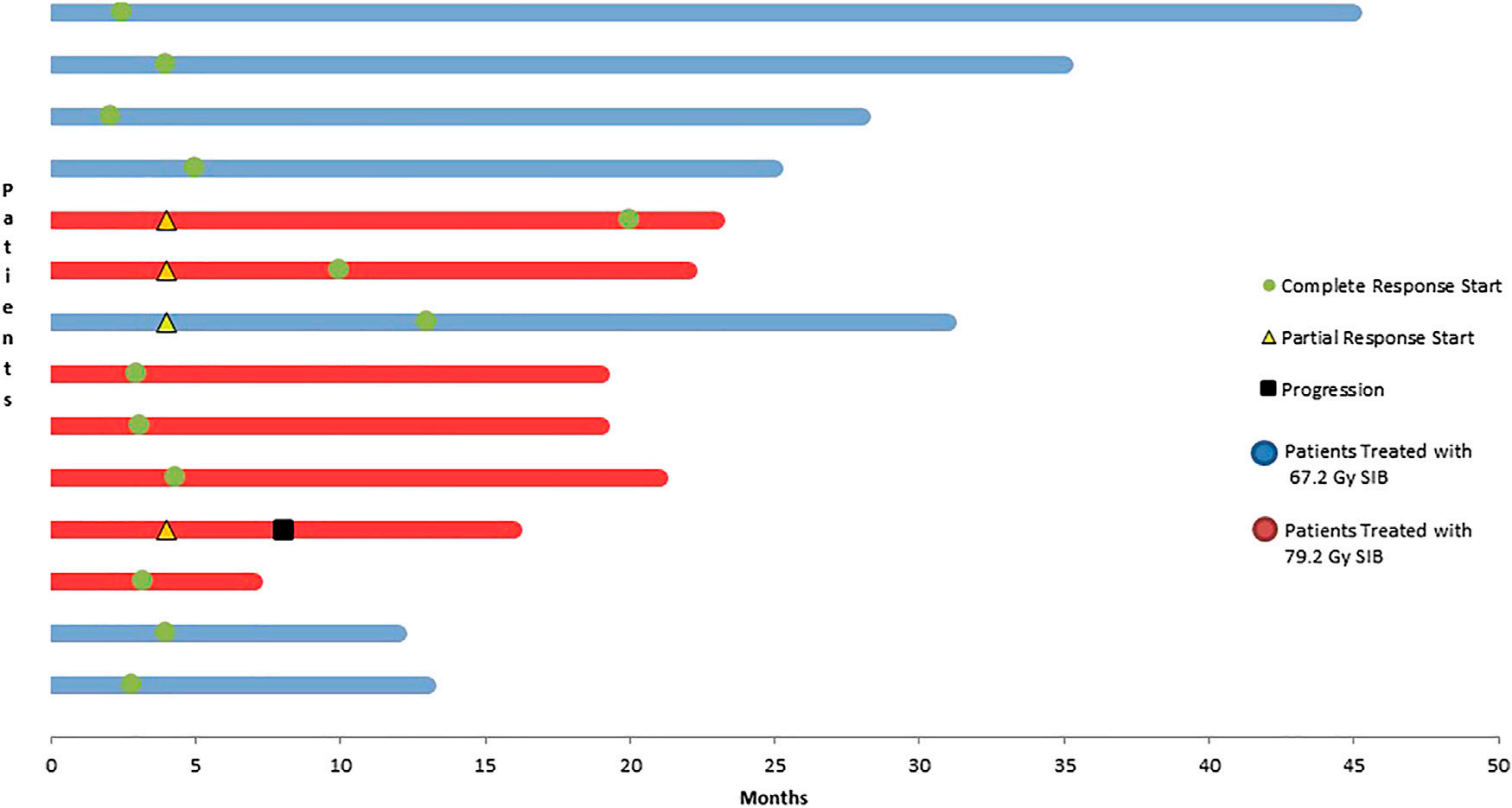
Evaluation of survival data at a median follow-up of 24 months.

At the termination of the follow-up, all of our patients were alive and symptom free. Follow-up visit was performed at 3 to 4 months after treatment termination. (Figure 5).

Complete remission was generally observed upon the first follow-up MRI at 3–4 months. In patients with higher tumor volumes receiving higher doses, complete remission occurred more slowly. In this population the first follow-up imaging showed only partial remission in 42% of patients. PET/CT, a diagnostic procedure complemented with biological information, was performed after 2 consecutively positive MRI results. It yielded to a negative result in 5 patients and only 1 patient had a positive result.

As clearly noted, all of our patients are alive. However, disease progression occurred in one patient due to local progression, and this patient received chemotherapy at the time of the termination of data collection.

## Discussion

A prerequisite of performing state-of-the-art image-guided and ultra-precision, ultra-conformal RT-s is the precise determination of the target volume. This information is of great importance for the use of definitive RT in cervical cancer and during a dose escalation study. The mandatory role of MRI and PET/CT scan for detecting the primary and lymph node or distant metastases is shown in several studies [2–4]. Compared to MRI, the sensitivity of PET/CT in detecting lymph node metastases was 50 and 83%, respectively. The lymph nodes confirmed as abnormal by histology but negative by PET were less than 1 cm in size [5].

Definitive radiation therapy for cervical cancers is performed in accordance with the following guidelines. The standard treatment in the therapy of this tumor type is the BT boost, which method has been continually improved since the 1960s. In the generally accepted protocols, a total dose of 45 to 50 Gy is delivered to the pelvic region. External beam RT is followed by 3 to 4 sessions of BT at a dose of 7 Gy, which is delivered as a single dose to the residual lesion using the HDR technique. For the determination of the residual tumor, MRI-controlled BT is the most effective method as suggested by Pötter et al. [2]. The delivery of the following doses is recommended to the designated target areas: 85 to 90 Gy EQD2 (D90) to CTV-T_HR_, 60 Gy (D98) to CTV-T_IR_, and 90 Gy (D98) to GTV_RES_-T. The coverage of the anatomically defined point “A” should be kept in mind at all times, which should be 75 Gy (EQD2). By following these dose requirements, maximum success in the treatment of the tumor can be achieved [2]. Since the tumor is surrounded by normal tissues, the dose limitations of these tissues must be kept in mind to avoid late toxicity. In the case of HDR BT, the following dose limits relating not to an entire organ but to 2 cc are respected: rectal wall D2cc, 64 Gy; bladder wall D2cc, 85 Gy; and sigmoid colon wall D2cc, 63 Gy [6]. The clinical outcome of MRI based image guided adaptive BT (IGABT) was observed by Pötter et al. Between 2001–2008 156 patients were treated with IGABT. In the early IB–IIB stages a 95–100% local control (LC) could be achieved at 3 years follow up period. In the more advanced stages (IIB/III/IV) LC rates were 85–90%. In this single-institutional study moderate treatment related toxicity was observed [7].

In the RetroEMBRACE multicenter trial over 700 patients with cervical cancer were included, for IGABT. The LC at 3/5 years for IB, IIB, IIIB was 98%/98%, 93%/91%, 79%/75%. Treatment related morbidity at 5 years was 5, 7, 5% for bladder, gastrointestinal tract, vagina [8]. More important of these studies were the results of QoL for BT treated patients. The early 24 months report from the EMBRACE study showed for vaginal morbidity no severe increase. Less than 3.6% of the patient reported > grade 3 vaginal complaints. Although grade I (89%) and grade II (29%) morbidity was present [9]. The QoL data from the EMBRACE study was observed with the questionnaires of EORTC QLQ-30, and CX-24 from all 744 patients, and were presented in 2015. The questionnaires were completed at the baseline, then every 3 months in the first year, and every 6 months in the second and third year. The general and functional QoL was impaired at the baseline then got better during the follow up period. Tumor related symptoms resolved after treatment, but treatment related symptoms developed or persisted after treatment ending [10].

Along with the development of external beam RT, other therapeutic options have also emerged. In addition to the application of the IMRT technique, several stereotactic treatment attempts were made to replace the known treatment algorithm of the tumor. Kilic et al. summarized the research in this field by reviewing publications in the PubMed database. During the review, retrospective studies were mostly found, but 3 prospective studies were also identified. These studies involved low numbers of patients and applied different techniques. Stereotactic boost or IMRT boost were generally performed instead of BT in those cases where either the patient refused the therapy or it was not feasible due to anatomical reasons. In these treatments, total doses of 16 to 36 Gy were delivered at doses of 1.8 to 6 Gy per fraction [1]. In the realm of intensity-modulated external beam RT, some authors dealt with the issue of replacement of BT in the treatment of cervical cancers. The advantageous features of the technique include better protection of organs at risk and homogenous coverage [11, 12].

The application of the SIB technique was also proposed by other authors as a possibility for external field dose escalation. Guerrero et al. studied cases of cervical cancer where a BT boost providing appropriate coverage could not be delivered due to the size of the tumor. In such cases, the simultaneous integrated boost (SIB) technique can, in theory, provide an appropriate alternative. Based on the linear quadratic model used in RT and the calculation of the biologically effective dose, the dose of external beam RT of the pelvis with added BT may be equal to the dose of the SIB technique. In their practice, the following doses were delivered: 25 x 3.1 Gy, −2.8 Gy, −2.4 Gy. Bladder and colon exposure did not exceed average doses of 60 and 70 Gy, respectively. The treatment time could be shortened to 5 weeks. In light of the above information, in our opinion, the SIB IMAT treatment technique offers a reassuring alternative to the conventional technique [13].

Vandecasteele et al. studied the feasibility of arc therapy in patients with inoperable cervical cancer. Based on PET/CT-based planning, the delivery of median doses (D50) of 62, 58, and 56 Gy to the primary tumor was recommended. The delivery of a 60 Gy (D98) dose to PET-positive lymph nodes was recommended. In the 9 studied cases, the IMAT technique made the delivery of the dose to the primary tumor and the positive lymph nodes by SIB possible [14].

Several studies reporting on the successful application of the SIB technique have been published recently. Wang et al. integrated a dose of 60.2 Gy delivered in 28 fractions into the total pelvic irradiation at a total dose of 50.4 Gy. The integrated boost technique was compared with a regimen of a 3-Gy boost delivered on 3 occasions following conventional fraction delivery (25 x 2 Gy). Both treatment alternatives provided excellent local control values (98 vs. 100%), and no late-onset toxicity was observed [15].

During their neoadjuvant study involving 30 patients, Vandecasteele et al. integrated the boost dose of 2.48 Gy into 25 fractions, which was delivered in addition to the total pelvic dose of 45 Gy. At the end of the treatment, during the 2-year follow-up period, local control of 96% was achieved. Late-onset adverse reactions were considered acceptable, as grade 4 intestinal toxicity occurred in 4% of the participants, and grade 3 urinary side effects occurred in 14% [16]. O’Donell et al used the National Cancer Database to study women with invasive cervical cancer who were treated with radiation between 2004 and 2013 either with BT or IMRT or stereotactic body radiotherapy (SBRT) boost. Outcomes were evaluated among 15,905 cervical cancer patients with Kaplan-Meier and propensity score matching. The propensity score match results showed significant difference for patients treated with BT boost than to IMRT boost patients. There was no significant difference for SBRT boost patients. The authors suggest the SBRT boost could be a therapy option [17]. Herrera et al found that SIB treatment for cervix cancer patient is promising but tumor motion should be taken in account to avoid target under-dosing and OAR over-dose [18]. In the study of Morgenthaler et al SBRT boost was performed to 31 cervical cancer patients with IB–IVB stage tumor on Cyberknife. The results were presented at a median follow-up of 40 months. No severe acute toxicity was observed and LC was 92% at 3 and 5 years. The median PFS was 41 months [19].

The role of brachytherapy is well known in the treatment of cervical cancer. The new development in treatment suggest a population of patient how could benefit of either SBRT or SIB/IMRT. To summarize the findings of these papers further investigation in well-designed prospective clinical studies is requested to find the relevant dose for both techniques.

In our own study, a similarly excellent local control rate was achieved with completely tolerable adverse reactions and an acceptable quality of life. With respect to the totality of patients, disease-free survival is good; however, it is difficult to evaluate this metric given the short follow-up period.

However, the limitations of our study are the small patient number and the two different treatment arms, which makes it difficult to interpret our data comparing to previous studies. Although, it strengthens the suggestion, as in other small case studies, for the use of a novel technical approach, SIB, in the treatment of cervical cancer patients with no option for BT.

## Conclusions

Based on our results, in patients with advanced cervical cancer who cannot receive brachytherapy boost, definitive external beam radiotherapy with an integrated boost replacing brachytherapy is feasible and may represent an appropriate alternative. Though the routine use of this treatment cannot be recommended yet due to the lack of well-powered comparative studies, our result and the favourable comparison with the standard treatment supports the further evaluation of this technique on a larger patient population.

## Data Availability

The datasets presented in this study can be found in online repositories. The names of the repository/repositories and accession number(s) can be found in the article/Supplementary Material.
